# Long-term follow-up in primary Sjögren’s syndrome reveals differences in clinical presentation between female and male patients

**DOI:** 10.1186/s13293-017-0146-6

**Published:** 2017-08-08

**Authors:** Jorge I. Ramírez Sepúlveda, Marika Kvarnström, Per Eriksson, Thomas Mandl, Katrine Brække Norheim, Svein Joar Johnsen, Daniel Hammenfors, Malin V. Jonsson, Kathrine Skarstein, Johan G. Brun, the DISSECT consortium, Agneta Zickert, Albin Björk, Anders A. Bengtsson, Andreas Jönsen, Andrei Alexsson, Anna Tjärnlund, Ann-Christine Syvänen, Antonella Notarnicola, Argyri Mathioudaki, Åsa Karlsson, Carin Backlin, Christine Bengtsson, Christopher Sjöwall, Dag Leonard, Daniel Hammenfors, Elisabet Svenungsson, Elke Theander, Eva Baecklund, Eva Murén, Fabiana Farias, Gerli Pielberg, Guðný Ella Thorlacius, Gunnel Nordmark, Hector Chinoy, Helena Andersson, Helena Enocsson, Helena Forsblad-d’Elia, Ingrid E. Lundberg, Iva Gunnarsson, Janine Lamb, Jennifer Meadows, Jessika Nordin, Johan G. Brun, Johanna Dahlqvist, Johanna K. Sandling, John Mo, Jonas Carlsson Almlöf, Jonas Wetterö, Jorge I. Ramírez Sepúlveda, Juliana Imgenberg-Kreuz, Karin Bolin, Karin Hjorton, Karl A. Brokstad, Karolina Tandre, Kathrine Skarstein, Katrine Brække Norheim, Kerstin Lindblad-Toh, Lars Rönnblom, Leonid Padyukov, Lilian Vasaitis, Lina Hultin-Rosenberg, Louise Pyndt Diederichsen, Maija-Leena Eloranta, Malin V. Jonsson, Marie Wahren-Herlenius, Marika Kvarnström, Maryam Dastmalchi, Matteo Bianchi, Niklas Hagberg, Outi Vaarala, Øyvind Molberg, Per Eriksson, Roald Omdal, Robert G. Cooper, Roland Jonsson, Sara Magnusson Bucher, Sergey Kozyrev, Silke Appel, Simon Rothwell, Solbritt Rantapää-Dahlqvist, Svein Joar Johnsen, Thomas Mandl, Lars Rönnblom, Helena Forsblad-d’Elia, Sara Magnusson Bucher, Eva Baecklund, Elke Theander, Roald Omdal, Roland Jonsson, Gunnel Nordmark, Marie Wahren-Herlenius

**Affiliations:** 1 Unit of Experimental Rheumatology, Department of Medicine, Karolinska University Hospital, Karolinska Institutet, SE-171 76 Stockholm, Sweden; 2 Division of Rheumatology, Department of Clinical Experimental Medicine, Linköping University, Linköping, Sweden; 3 Department of Rheumatology, Skåne University Hospital, Malmö, Sweden; 4 Clinical immunology unit, Department of Internal Medicine, Stavanger University Hospital, Stavanger, Norway; 5 Broegelmann Research Laboratory, Department of Clinical Science, University of Bergen, Bergen, Norway; 6 Department of Rheumatology, Haukeland University Hospital, Bergen, Norway; 7 Section for Oral and Maxillofacial Radiology, Department of Clinical Dentistry, University of Bergen, Bergen, Norway; 8 Gade Laboratory for Pathology, Department of Clinical Medicine, University of Bergen, Bergen, Norway; 9 Department of Pathology, Haukeland University Hospital, Bergen, Norway; 10 Department of Medical Sciences, Rheumatology and Science for Life Laboratory, Uppsala University, Uppsala, Sweden; 11 Department of Public Health and Clinical Medicine, Rheumatology, Umeå University, Umeå, Sweden; 12 Department of Rheumatology, Faculty of Medicine and Health, Örebro University, Örebro, Sweden

**Keywords:** Sjögren’s syndrome, Autoimmunity, Sex difference, Disease severity, Extraglandular manifestations

## Abstract

**Background:**

Despite men being less prone to develop autoimmune diseases, male sex has been associated with a more severe disease course in several systemic autoimmune diseases. In the present study, we aimed to investigate differences in the clinical presentation of primary Sjögren’s syndrome (pSS) between the sexes and establish whether male sex is associated with a more severe form of long-term pSS.

**Methods:**

Our study population included 967 patients with pSS (899 females and 68 males) from Scandinavian clinical centers. The mean follow-up time (years) was 8.8 ± 7.6 for women and 8.5 ± 6.2 for men (ns). Clinical data including serological and hematological parameters and glandular and extraglandular manifestations were compared between men and women.

**Results:**

Male patient serology was characterized by more frequent positivity for anti-Ro/SSA and anti-La/SSB (*p* = 0.02), and ANA (*p* = 0.02). Further, men with pSS were more frequently diagnosed with interstitial lung disease (*p* = 0.008), lymphadenopathy (*p* = 0.04) and lymphoma (*p* = 0.007). Conversely, concomitant hypothyroidism was more common among female patients (*p* = 0.009).

**Conclusions:**

We observe enhanced serological responses and higher frequencies of lymphoma-related extraglandular manifestations in men with pSS. Notably, lymphoma itself was also significantly more common in men. These observations may reflect an aggravated immune activation and a more severe pathophysiological state in male patients with pSS and indicate a personalized managing of the disease due to the influence of the sex of patients with pSS.

## Background

It has been widely established that women are more prone to develop autoimmune diseases [1]. Primary Sjögren’s syndrome (pSS) is a systemic autoimmune disease characterized by inflammation of the salivary and lacrimal glands, causing a reduction in exocrine secretion that ultimately leads to the clinical presentation of sicca symptoms. The reported population-based female to male ratio is 14:1 [2]. Many hypotheses have been proposed to explain the overall marked sex bias in autoimmunity and pSS [3], including genetic and epigenetic factors [4, 5], sex hormones [6], and X-chromosome aberrances [7, 8]. However, the molecular mechanisms that drive this sex skewing still remain elusive.

Interestingly, the differences between the sexes likewise extend to the clinical manifestations, where female and male patients differ in disease presentation and severity. Despite being less prone to develop autoimmune diseases, male patients have been reported to have a more severe disease and a worse prognosis. In systemic lupus erythematosus (SLE), men present more renal disease [9] and serositis [10]. Further, Andrade et al. [11] identified male sex as a strong predictor for poorer long-term prognosis due to accelerated damage accrual, while Manger et al. [12] reported male sex as a risk factor for increased SLE mortality. Male sex is also deemed as a factor for accelerated disease progression in multiple sclerosis (MS) [13] and associated with a significantly higher prevalence of comorbidities [14] such as diabetes, epilepsy, depression, and anxiety. Although less clear, sex differences in rheumatoid arthritis (RA) severity and extra-articular manifestations have also been described; women appear more prone to present sicca symptoms and men to have erosive joint disease, rheumatoid nodules, and interstitial lung disease [15, 16].

Regarding pSS, several studies have addressed sex differences in clinical presentation [17, 18]. As reviewed by Brandt et al. [3], some authors have identified differences in extraglandular manifestations and serological markers, with a focus on female prevalence. However, there is no clear consensus on whether male sex is associated with a more severe disease. Our group has described that at diagnosis, male patients more frequently present with extraglandular manifestations, more concomitant extraglandular manifestations, and higher anti-Ro52 levels by investigation in two independent cohorts [19]. To understand if there are sex differences in the clinical presentation of pSS also after a long-standing disease and whether risk for comorbidities vary between the sexes, we assessed glandular and extraglandular manifestations, serological parameters, and comorbidities of pSS in men and women years after diagnosis in a large Scandinavian cohort.

## Methods

### Patients

DISSECT—“Dissecting disease mechanisms in three systemic inflammatory autoimmune diseases with an interferon signature”—is a multicenter consortium comprising the Scandinavian Sjögren’s syndrome research network, the Swedish SLE network and the Swedish Myositis network linked to the European Myositis network. All 967 patients with pSS and fulfilling the American–European consensus criteria [20] in the DISSECT cohort were included in this study. Out of these, 899 were females and 68 were males (Table 1). The patients were diagnosed and followed at the Departments of Rheumatology at the University Hospitals in Gothenburg, Skåne, Linköping, Örebro, and Uppsala, as well as the Karolinska University Hospital in Stockholm, Sweden, and the Department of Rheumatology at Haukeland University Hospital, Bergen, and the University Hospital in Stavanger, Norway. Of the 205 patients from the Karolinska University Hospital, 127 were included in a previous analysis of clinical manifestations in female and male patients at diagnosis [19]. Clinical data with regard to autoantibody status and clinical manifestations were retrieved from the patient’s medical records. This included information on sicca symptom onset, age at diagnosis, histopathological examination of minor labial salivary gland biopsies, and serological analysis of ANA, Ro/SSA, and La/SSB autoantibodies. ANA was determined by indirect immunoflourescence of Hep2 cells for the vast majority of patients, while methods for determining Ro/SSA and La/SSB autoantibodies at the respective accredited Clinical Immunology department varied over time and between the centers and included indirect immunofluorescence of transfected cells, immunoblotting, ELISA, and multiplex technologies. Information on extraglandular manifestations according to doctors’ clinical assessments included articular, pulmonary, renal, cutaneous, muscular, endocrine, and lymphoid systems. The study was approved by the local ethical committee for respective study center, and patients gave informed written consent.

**Table 1:** Demographic and basic characteristics of the cohort

	Women *n* = 899% (frequency)	Men *n* = 68% (frequency)	*p* value
Basic characteristics			
Sex	93% (899/967)	7% (68/967)	*<0.0001*
Age at symptom onset (years, mean ± SD)	46.16 ± 14.79	47.88 ± 14.71	0.50
Age at diagnosis (years, mean ± SD)	52.65 ± 13.75	52.63 ± 13.52	0.96
Follow-up time from diagnosis (years, mean ± SD)	8.76 ± 7.62	8.48 ± 6.15	0.68
Item IV. Histopathology^a^			
Salivary gland biopsy			
Performed	87% (760/871)	79% (52/66)	0.06
Positive (focus score ≥1)	90% (616/685)	86% (42/49)	0.35
Germinal centers	21% (75/351)	33% (8/24)	0.20

Bold values indicate statistically significant findings (*p* < 0.05)

^a^According to the 2002 Revised American-European Consensus Group criteria for Sjögren’s syndrome

### Statistical analysis

For the comparison of continuous variables, the Mann–Whitney *U* test was used. The chi-square test was used when analyzing categorical data, and Fisher’s exact test was employed if the observed frequency of any given cell was <5 and/or the total number of analyzed individuals in any group was <40. Data was analyzed with GraphPad Prism 6, and *p* values <0.05 were considered statistically significant.

## Results

### Basic characteristics of the cohort

The cohort consisted of 967 pSS patients, of which 899 were women (93%) and 68 were men (7%) (Table 1). The female/male ratio was 13:1. The mean age at symptom onset for the female group was 46 years ± 14.8 (95% CI) and 48 years ± 14.7 (95% CI) for the male group. There was no significant difference with regard to the age at diagnosis between women and men (52.6 and 52.6 years, respectively), or the follow-up time from diagnosis between female and male patients (8.8 years ± 7.6, 95% CI and 8.5 years ± 6.1, 95% CI, respectively).

We also compared the histopathological parameters of the salivary gland biopsy from all the included patients (Table 1). Although women tended to more frequently undergo a salivary gland biopsy than men (*p* = 0.06), the histological findings revealed no significant differences in terms of a positive focus score or presence of germinal center-like structures.

### Serological differences between female and male patients with pSS

Autoantibody profiles were also analyzed in a sex-specific manner (Table 2). Autoantibody positivity was defined as presenting both or either of anti-Ro/SSA or anti-La/SSB. Accordingly, 72% of the patients from this cohort were autoantibody positive; 71% of the female and 81% of the male patients had either Ro/SSA and/or La/SSB antibodies.

**Table 2:** Serological characteristics in female and male patients with pSS

	Women *n* = 899% (frequency)	Men *n* = 68% (frequency)	*p* value
Item VI.^a^ Autoantibodies			
Ro/SSA and/or La/SSB positive	71% (640/899)	81% (54/67)	0.1
Ro/SSA positive	68% (612/897)	76% (51/67)	0.18
La/SSB positive	41% (367/894)	57% (38/67)	*0.01*
Ro/SSA and La/SSB positive	38% (342/892)	52% (35/67)	*0.02*
ANA positive	74% (660/895)	87% (58/67)	*0.02*

Italicized values indicate statistically significant findings (*p* < 0.05)

^a^According to the 2002 Revised American-European Consensus Group criteria for Sjögren’s syndrome

Anti-Ro/SSA positivity was observed in 68% of the women and 76% of the men; SSB autoantibodies were detected in 41% of the women and 57% of the men (*p* = 0.01) and positivity for both anti-Ro/SSA and anti-La/SSB was found in 38% of the women and 52% of the men (*p* = 0.02) (Table 2). Furthermore, ANA positivity was significantly more frequent in male patients (*p* = 0.02). Thus, the stratified analysis indicated that the male group presents significantly higher frequencies of positivity towards ANA, La/SSB, and Ro/SSA + La/SSB.

**Fig. 1:**
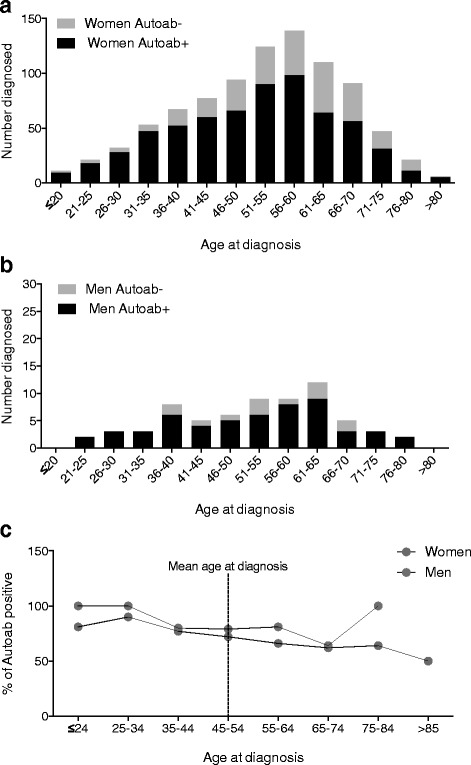
Ro/SSA- and/or La/SSB-positive and negative women and men diagnosed with Sjögren’s syndrome at different age intervals. **a** Number of Ro/SSA- and/or La/SSB-positive and negative women diagnosed with Sjögren’s syndrome. **b** Number of Ro/SSA and/or La/SSB positive and negative men diagnosed with Sjögren’s syndrome. **c** Percentage of Ro/SSA and/or La/SSB positive women and men diagnosed with Sjögren’s syndrome

Sex hormones have been suggested to influence the immune system, especially in terms of antibody production [21]. To evaluate whether the number or percentage of autoantibody positive individuals diagnosed was related to menopause, we further stratified the female and male patients with and without autoantibodies based on age at diagnosis (Fig. 1). We observed an increasing number of autoantibody-positive women being diagnosed up to 60 years of age, and that at the same time, a steadily increasing number of autoantibody negative women receiving the diagnosis (Fig. 1a). The male group displayed a comparable pattern (Fig. 1b). Consistently, also when analyzed as percent autoantibody positive (Fig. 1c), the trend was similar in both the female and male groups. Already in the late thirties/early forties the percentage of autoantibody-positive patients diagnosed with pSS started to decline and did so steadily until the mid-seventies. Very few males were diagnosed after the age of 75 (*n* = 2), making the last point of the curve less relevant to consider. Altogether, the data show a consistent higher percentage of autoantibody-positive men. Neither the female nor male group did show any obvious change specific for age 50 or 55, which is commonly used as a proxy for menopause.

### Frequencies of clinical manifestations differ between female and male pSS patients

The presence and type of extraglandular manifestations were obtained in order to evaluate differences in frequencies among the sexes. There were significant sex differences in the frequencies of several extraglandular manifestations (Table 3). Interstitial lung disease was more frequent in male patients (*p* = 0.008), as well as lymphadenopathy (*p* = 0.04) and, notably, lymphoma (*p* = 0.007). There was also a tendency for men to present more often with major salivary glands swelling (*p* = 0.11), as well as myositis (*p* = 0.14), while hypothyroidism was more common in female patients, present in 24% of the women as opposed to 8% of the male patients (*p* = 0.009).

**Table 3:** Frequency of pSS-associated clinical manifestations and comorbidities in female and male patients with pSS

	Women *n* = 899% (frequency)	Men *n* = 68% (frequency)	*p* value
Classification^a^			
Articular			
Arthritis	20% (169/859)	14% (9/65)	0.25
Pulmonary			
Interstitial lung disease	6% (40/619)	17% (8/48)	*0.008*
Renal			
Interstitial nephritis	3% (19/607)	2% (1/48)	1.00
Cutaneous			
Dermal vasculitis	11% (91/836)	8% (5/62)	0.49
Lymphadenopathy and lymphoma			
Enlarged lymph nodes	8% (69/825)	16% (10/62)	*0.04*
Lymphoma	4% (32/889)	10% (7/68)	*0.007*
Muscular			
Myositis	0.9% (7/772)	3% (2/61)	0.14
Glandular			
Major salivary gland swelling	29% (225/767)	40% (21/53)	0.11
Presence of EGM	34% (304/899)	41% (28/68)	0.22
Number of EGM (mean + SD)	0.44 ± 0.69	0.51 ± 0.74	0.30
Other common comorbidities and clinical manifestations			
Hypothyroidism	24% (175/739)	8% (4/51)	*0.009*
Raynaud’s phenomenon	29% (247/851)	30% (20/66)	0.83

Italicized values indicate statistically significant findings (*p* < 0.05)

^a^Available data on the extraglandular manifestations evaluated to estimate the EULAR Sjögren’s Syndrome Disease Activity Index (ESSDAI) are included

*EGM* extraglandular manifestations

Considering the observation that men with pSS present an increased risk for lymphoma when compared to women (Table 3), we analyzed the difference between histopathologically verified subtypes of lymphoma. Mucosa-associated lymphoid tissue (MALT) lymphoma and diffuse large B cell lymphoma (DLBCL) were the most common subtypes, but no significant difference between the occurrence between women and men was found (Table 4).

**Table 4:** Frequencies of subtypes of lymphoma in women and men with pSS

	Women *n* = 32% (frequency)	Men *n* = 7% (frequency)	*p* value^a^
MALT lymphoma	56% (18/32)	57% (4/7)	1.00
DLBCL	16% (5/32)	0% (0/7)	0.56
Follicular lymphoma	6% (2/32)	14% (1/7)	–
Myeloma	3% (1/32)	0% (0/7)	–
CLL	3% (1/32)	0% (0/7)	–
Lymphoplasmacytic lymphoma	0% (0/32)	14% (1/7)	–
Other NHL	13% (4/32)	14% (1/7)	–
Hodgkin lymphoma	3% (1/32)	0% (0/7)	–

According to the WHO 2016 classification

^a^Calculated when *n* ≥ 5 for either group

*MALT* mucosa-associated lymphoid tissue, *DLBCL* diffuse large B cell lymphoma, *CLL* Chronic lymphatic leukemia, *NHL* non-Hodgkin lymphoma

### Autoantibody-positive pSS female and male patients differ in terms of clinical manifestations

We further assessed whether presentation between autoantibody-positive female and male patients differed from the unstratified analysis described above, focusing on the extraglandular manifestations and other clinical manifestations that had significantly differed between female and male patients regardless of serology-status. Interstitial lung disease and lymphoma were significantly more frequent in men (*p* = 0.01 and *p* = 0.03, respectively) also when including only seropositive cases in the analysis, and hypothyroidism was more common in women (*p* = 0.03) (Table 5). Lymphadenopathy had a higher observed frequency among male patients (Table 3), which was however not statistically significant when considering only the autoantibody positive cases.

**Table 5:** Frequency of comorbidities, extraglandular, and other common clinical manifestations in SSA- and/or SSB-positive female and male patients with pSS

	Women *n* = 640% (frequency)	Men *n* = 54% (frequency)	*p* value
Interstitial lung disease	7% (31/444)	19% (7/37)	*0.01*
Enlarged lymph nodes	10% (57/584)	16% (8/49)	0.15
Hypothyroidism	23% (117/517)	8% (3/40)	*0.03*
Lymphoma	4% (28/634)	11% (6/54)	*0.03*

Italicized values indicate statistically significant findings (*p* < 0.05)

## Discussion

Primary Sjögren’s syndrome represents the autoimmune disease with the highest female bias, ranging from a ratio of 10–20:1 [22]. Besides the overwhelming sex bias observed in disease susceptibility, previous studies have also aimed to investigate whether the disease manifests differently between female and male patients. Earlier observations have not reached a clear consensus as to whether male pSS patients have a distinct clinical course and a more severe presentation of the disease [3]. In a recent study, though, we reported that men with pSS, from a population-based incident case cohort, displayed significantly higher levels of anti-Ro52, were diagnosed at an earlier age than their female counterparts, presented more concomitant extraglandular manifestations, and had a higher frequency of pulmonary complications and cutaneous vasculitis. Similarly, an Italian cohort revealed a significant male propensity towards extraglandular manifestation presentation [19]. These findings strongly suggest that men affected by pSS have a more severe disease at time of diagnosis. In the present study, we addressed whether differences between the sexes are also present several years after diagnosis.

We identified significant sex differences in terms of serological parameters and frequencies of some organ involvement. Our results indicate that the humoral response between women and men is different; particularly, men present more often with La/SSB, Ro/SSA + La/SSB and ANA positivity. This increased immune activity observed among the male patients is of special interest since in a healthy state, men mount a lower antibody response in comparison with women [23–25]. Although the pathogenic effect of autoantibodies has not been clearly established, the presence of certain autoantibodies has been associated with organ manifestations. Noteworthy, SSA antibodies are related to pulmonary diseases [26, 27], an extraglandular manifestation we observed overrepresented in the male patients from our cohort. Further, a recent study proposed that anti-La/SSB antibodies are a risk factor associated with increased mortality in pSS patients [28]. Thus, even though the pathogenic role of pSS-associated autoantibodies remains unknown, seropositivity has a strong correlation with organ involvement and worse prognosis, supporting the conclusion that the disease course is more severe in male patients than in female patients.

Interstitial lung disease has been extensively studied in the context of pSS [29, 30]. Male sex is widely recognized as a risk factor for developing interstitial lung disease [26, 31]. In accordance with more recent studies [19], our extended cohort shows that male patients with pSS are indeed more prone to develop interstitial lung disease. The reasons for this male preponderance are poorly understood; however, this might be due to increased seropositivity, environmental exposure to certain pollutants [32] and smoking [31] in the male pSS group. In fact, idiopathic pulmonary fibrosis, which represents a usual interstitial pneumonia histopathological pattern, is one of the most common forms of interstitial lung disease detected in pSS [29] and has a higher prevalence in men, as epidemiological studies have previously described [33, 34]. In other words, regardless of pSS diagnosis, men in general are more frequently affected by a type of interstitial lung disease that is associated with a worse prognosis. This susceptibility, thus, might be augmented in pSS, driven by other pathophysiological factors that enhance this propensity to develop pulmonary disease.

It is well known that pSS patients have an increased risk for developing non-Hodgkin lymphoma [35–38]. Sex-specific risk for lymphoma development in patients with rheumatic disease has been seldom studied, mainly due to the inclusion of mostly female patients. Nevertheless, Ansell et al. have reported a significantly higher incidence of lymphoma in male RA patients. Despite the increased association of autoimmune diseases and lymphoma in men [39], earlier studies of sex differences in lymphoproliferative malignancies in pSS have not shown a clear sex-specific predominance [40, 41]. In contrast, our present study is the largest pSS cohort to report a significantly increased risk for male patients to present lymphoma in comparison with female patients. This is in accordance with the results from a smaller patient sample from which an increased risk for men affected with pSS, SLE, RA, and autoimmune hemolytic anemia to develop lymphoma was reported [42].

Since the male bias observed in pSS-associated malignancies has only recently been described, it is not fully understood. However, considering the reported predictive factors of lymphoma development, an increased risk of lymphoma in male patients is logical. As reviewed by Nocturne and Mariette [43], the main clinical manifestations and parameters associated with this type of cancer are swelling of salivary glands, lymphadenopathy, palpable purpura, cryoglobulinemia, lymphopenia, low complement levels, and a monoclonal component in serum or urine. Interestingly, the male pSS patients from our cohort presented more frequently with lymphadenopathy. Although not included in the data analysis due to the high amount of missing data, cryoglobulinemia was also more commonly observed in the male patients (20/151 in females vs 3/7 in males, *p* = 0.03).

The only clinical manifestation that was more significantly represented in the female pSS patients from our cohort was hypothyroidism. Endocrine problems are not uncommon in patients with autoimmune disorders [44], and the female bias towards thyroid diseases has been extensively documented [45–48]. Furthermore, since the predominantly female incidence of hypothyroidism corresponds with the female susceptibility to autoimmune diseases, the thyroid gland has been proposed as a decisive organ to explain the sex skewness in autoimmune diseases. The effect of adipokines, which comprise a number of different cytokines including, e.g., leptins, adiponectins, TNF-α, and IL-6, on thyroid tissue has been suggested as a triggering mechanism for autoimmune thyroiditis, which probably precedes or coincides with the diagnosis of another systemic autoimmune disease such as pSS and SLE. [44]. This emphasizes the need for a comprehensive screening and close monitoring of thyroid function in suspected patients because it might be an important marker for autoimmune disease development. As for the thyroid gland being responsible for female-preponderant diseases, further studies should be performed to clarify its role.

The studied cohort offers a valuable large group of clinically carefully characterized patients with pSS, allowing for analysis of parameters that differ between men and women affected by the syndrome. The long follow-up time is essential for identifying clinical manifestations at different time points of the disease course. However, the patients included in this cohort were mostly included at tertiary referral centers of university hospital clinics. A possible limitation of the study is that the study population might therefore not mirror the general pSS patient population and that the patients described in this study represent cases with an overall more severe disease phenotype, both female and male patients. A further possibility is that male patients with mild symptoms and less severe disease are less often referred, as the primary health care doctor may not be as likely to suspect Sjögren’s syndrome due to its rarity in men, resulting in only men with more severe disease being included in the study. As the evaluation of extraglandular manifestations was dependent on doctors’ clinical assessments and did not include specific laboratory or physiologic tests unless the patient had symptoms, it is also possible that subclinical extraglandular manifestations may have been missed. However, the mean number of extraglandular manifestations diagnosed did not differ significantly between centers, nor did the proportion of men and women contributed. A further limitation is the lack of EULAR primary Sjögren’s syndrome disease activity (ESSDAI) and patient-reported (ESSPRI) indexes [49] at diagnosis, as well as information on other common extraglandular manifestations such as neurological diseases.

## Conclusions

In summary, our findings provide compelling evidence that the clinical presentation of pSS differs between women and men. The sex-specific preference of some clinical manifestations hints at divergent pathophysiological mechanisms between women and men with pSS. Consequently, management of the disease will benefit from sex-specific tailored clinical programs to address complications that are common or expected in the respective sex.
